# Nodular Goiter as the Presenting Symptom of DICER1 Tumor Predisposition Syndrome

**DOI:** 10.7759/cureus.77621

**Published:** 2025-01-18

**Authors:** Drew Burgess, Felicia Cooper, Rhea Birusingh, Shilpa Gurnurkar

**Affiliations:** 1 Harriet L. Wilkes Honors College, Florida Atlantic University, Boca Raton, USA; 2 Pediatric Endocrinology, Nemours Children's Hospital, Orlando, USA; 3 Pathology, Nemours Children's Hospital, Orlando, USA

**Keywords:** dicer1, goiter, pediatric, thyroid, tumor

## Abstract

In the context of pediatric thyroid disorders, a goiter may serve as an indication of a thyroid tumor in rare instances. In even more unusual scenarios, the cancer can exhibit features of two different categories of carcinoma types. This presentation may suggest a familial tumor syndrome. We present a previously healthy child with an unusual thyroid tumor histology that led to genetic testing and a diagnosis of DICER1 syndrome, an incredibly rare genetic disorder.

## Introduction

Thyroid carcinomas are uncommon in the pediatric population. Papillary thyroid cancers account for 85% of childhood thyroid carcinomas and tend to have excellent prognosis [1]. Invasive encapsulated follicular variant of papillary thyroid carcinoma (PTC) exhibits an increased likelihood of metastasizing in the blood vessels [2]. When the histological features of a thyroid carcinoma fall between two categories, such as classic papillary and follicular carcinoma, genetic testing can be instrumental in evaluating the patient’s predisposition to tumor development [2]. A rare hybrid tumor type, as well as the detection of certain proteins in the cell nuclei, may prompt investigation for a familial tumor syndrome. For example, DICER1 syndrome may be revealed, which is a genetic tumor syndrome caused by a mutation in the DICER1 tumor suppressor gene [3]. DICER1 syndrome’s hallmark is pituitary blastoma, but multinodular goiter and thyroid carcinoma can also result from this multiorgan disorder [4,5]. DICER1 syndrome is incredibly rare, with an estimated prevalence of less than one in 100,000 individuals and one in 4,600 in the oncologic population [6].

## Case presentation

**Figure 1:**
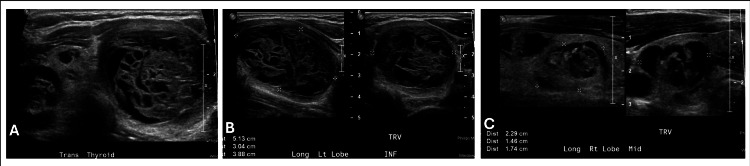
First thyroid ultrasound A. Transverse view of the thyroid gland showing a multinodular goiter; B. Left thyroid lobe nodules, the largest of which measures 5.2 cm; C. Right thyroid nodules, the largest of which measures 2.0 cm

**Figure 2:**
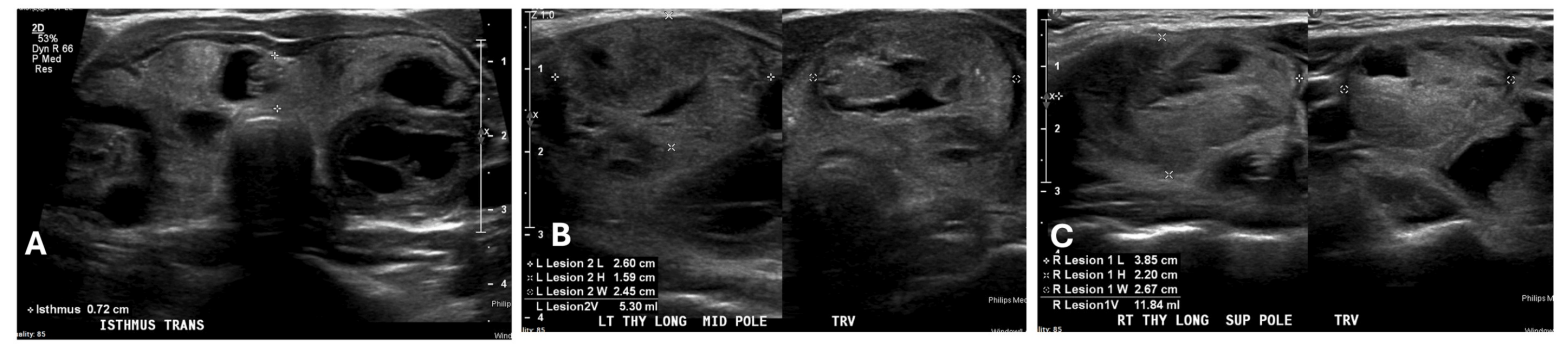
Second thyroid ultrasound A. Transverse view of the thyroid gland showing a multinodular goiter; B. Left thyroid lobe nodules, the largest of which measures 2.6 cm; C. Right thyroid lobe nodules, the largest of which measures 3.8 cm

An 11-year-old female patient with no past medical history presented to her primary care pediatrician with one to two weeks of neck swelling with no other symptoms. The patient lived in Florida, USA, an iodine-rich location. She had a paternal grandmother with Hashimoto's thyroiditis but otherwise family history was unremarkable. The patient denied dysphagia, dyspnea, and dysphonia. Upon examination, her pediatrician noted an enlarged thyroid, and labs and ultrasound were ordered. Diagnostic imaging revealed a multinodular goiter: three nodules in the left thyroid lobe and five nodules in the right thyroid lobe (Figure 1). The description and sizes of the nodules on thyroid ultrasound are detailed in Table 1. She was referred to our endocrinology clinic, where further evaluation indicated normal thyroid function tests with negative thyroid antibodies. A fine needle aspiration (FNA) of five of the nodules was performed to assist with preoperative risk stratification and planning of the extent of surgery/lymph node dissection. The nodules were all reported to be benign, Bethesda category II. Repeat serial thyroid ultrasounds were obtained twice yearly; 13 months after the initial FNA, some nodules increased, and some nodules decreased in size (Table 1, Figure 2). Another FNA was performed, and four nodules were biopsied. Four samples in each lobe were reported to have follicular cells of unknown significance (two samples graded Bethesda II and two samples graded Bethesda III). The patient underwent a total thyroidectomy. Additionally, enlarged lymph nodes were noted during surgery, and level VI lymph nodes were excised and sent to pathology. Postoperative thyroglobulin was 0.3 ng/ml, and thyroglobulin was checked approximately every three months; values ranged from 0.1 to 0.7 ng/ml. Thyroglobulin antibody and anti-thyroid peroxidase antibody were negative. The tumor, node, metastasis (TNM) staging was pT3a (tumor greater than 4 cm in greatest dimension limited to the thyroid), pN0 (one or more cytologically or histologically confirmed benign lymph nodes), pMx (not applicable, pM could not be determined from the submitted specimen).

**Table 1:** Thyroid ultrasound results

Ultrasound #1			
Nodule #	Region	Measurement	Description
1	Upper left	1 x 1.2 x 0.8 cm	Cystic/solid
2	Middle left	1.2 x 1.4 x 1 cm	Cystic/solid
3	Lower left	5.2 x 4.1 x 4 cm	Cystic/solid
4	Upper right	0.9 x 1.1 x 0.9 cm	Cystic/solid
5	Middle right	1.7 x 1.4 x 0.9 cm	Cystic/solid
6	Middle right	2 x 1.4 x 1.1 cm	Cystic/solid
7	Lower right	0.7 x 0.7 x 0.4 cm	Cystic/solid
8	Lower right	1.2 x 1.2 x 1.2 cm	Cystic/solid
Ultrasound #2			
Nodule #	Region	Measurement	Description
1	Upper left	2.0 x 1.6 x 1.6 cm	Cystic
2	Middle left	2.6 x 2.5 x 1.6 cm	Cystic
3	Lower left	2.3 x 1.7 x 2.2 cm	Spongiform
4	Upper right	3.8 x 2.7 x 2.2 cm	Cystic/solid
5	Middle right	No comment	
6	Middle right	2.5 x 1.8 x 2.5 cm	Cystic/solid
7	Lower right	2.2 x 1.9 x 1.4 cm	Cystic/solid
8	Lower right	No comment	

**Figure 3:**
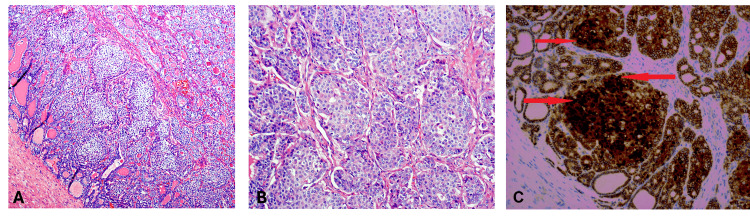
Histology of thyroid tissue obtained during total thyroidectomy A. Medium-power view showing infiltrative growth of squamous morules of tumor cells (hematoxylin and eosin stain); B. High-power magnification view of solid areas of tumor with cribriform pattern (hematoxylin and eosin stain); C. High-power magnification view showing diffuse and strong nuclear and cytoplasmic staining of infiltrating tumor cells (beta-catenin immunohistochemical stain)

The TNM staging classified the patient in the American Thyroid Association (ATA) pediatric low-risk category. Due to the unusual appearance of the lesion (Figure 3), with squamoid morulae, the final pathology was deferred to expert consultation at the University of Pennsylvania. The tumor was classified as an encapsulated follicular variant of papillary thyroid carcinoma (FV-PTC) with focal capsular invasion, mixed with areas comprising approximately 25% of the tumor exhibiting a cribriform-morular pattern, accompanied by nuclear beta (β)-catenin staining. The tumor was located in the right lobe of the thyroid, while both thyroid lobes displayed multinodular adenomatous goiter with focal papillary hyperplasia. No evidence of tumor necrosis or vascular invasion was observed. A total of 11 lymph nodes were examined, all of which were negative for tumor involvement. Due to the tumor’s unusual appearance as well as the strong nuclear and cytoplasmic β-catenin staining (indicating a possible underlying Wnt/β-catenin pathway activation), the patient was referred to the genetics department. Genetic testing revealed a pathogenic mutation in the DICER1 gene c.2531_2532del, confirming the diagnosis of DICER1 syndrome (Table 2). The patient now has periodic abdominal and pelvic ultrasound examinations (which have been unremarkable) and is monitored by the oncology and endocrinology departments due to the predisposition to develop other rare tumors. Thyroid ultrasounds are performed approximately every three to six months for surveillance. 

**Table 2:** Invitae thyroid cancer genetics panel results

Gene	Result
CHEK2	Negative
DICER1	Positive heterozygosity variant c.2531_2532del (p.Glu844Valfs*17)
APC	Negative
PRKAR1A	Negative
PTEN	Negative
RET	Negative
TP53	Negative

## Discussion

According to guidelines from the ATA, thyroid ultrasound may routinely be performed in children at increased risk of thyroid cancer (history of head and neck radiation, family cancer syndromes, etc.) and those with palpable nodules, thyroid asymmetry, and/or abnormal cervical lymphadenopathy on physical examination [7]. Unlike in guidelines for adults, nodule size is not as much a driving factor for FNA in children as the features of the nodule on imaging (such as microcalcifications, hypoechogenicity, or irregular margins). If the nodules are benign on FNA (as in our patient's first FNA), serial ultrasounds should be obtained with repeat FNAs if the nodules continue to grow or develop abnormal radiologic features. Because pediatric PTC is more likely to be bilateral or multifocal, a total thyroidectomy is recommended in most cases. If there is evidence of central and/or lateral neck metastasis, central neck dissection also should be conducted with the thyroidectomy. There is no single postoperative staging system validated for children with papillary thyroid cancer. The TNM system is limited in determining prognosis in children but excellent for describing disease extent and suggesting management. Based on TNM, the ATA has divided pediatric thyroid cancer into three risk categories (low risk, medium risk, and high risk) depending on regional lymph node and distal metastasis. The risk level also determines the thyroid-stimulating hormone (TSH) goal and frequency of laboratory monitoring (thyroglobulin, TSH-stimulated thyroglobulin, 123I scan, etc.). In general, a lower-risk thyroid cancer has a better prognosis and the lowest chance for distal metastasis. Our patient was classified as ATA pediatric low-risk due to the N0-classified lymph node invasion. Based on her category, the ATA recommends postoperative unstimulated thyroglobulin, a neck ultrasound six months postoperatively and then annually for the first five years, and thyroglobulin testing every three to six months for two years and then annually [7]. Thyroglobulin values are a marker of residual or recurrent thyroid carcinoma [7]. Due to the low-risk category and suppressed thyroglobulin values in our patient, she was not a candidate for radioactive iodine treatment according to the ATA.

There are four main types of thyroid cancer, varying in aggression and classified based on the cell types the cancer originated from: papillary, follicular, medullary, and anaplastic. Papillary and follicular thyroid cancers account for about 95% of thyroid carcinoma cases in both children and adults [8]. Papillary thyroid carcinoma has multiple subtypes, but they all exhibit “well-formed papillae lined by tumor cells” with an enlarged nucleus, irregular contours forming grooves, and chromatin in the peripheral margins [2]. Another feature of PTC is its predisposition for lymphatic metastasis. Common PTC subtypes include classical, follicular variant, and tall cell variants. While the classical subtype is associated with a good prognosis, the tall cell variant of PTC is more aggressive and has a worse prognosis; some researchers attribute the poor prognosis to mutations in the B-Raf proto-oncogene rather than its tall columnar cells and larger tumor size [9]. This subtype is commonly reported in older patients, with the mean age of presentation occurring between 41 and 66 years [9].

Like PTC, follicular thyroid carcinoma (FTC) contains cells that exhibit follicular differentiation; however, they lack the nuclear features of PTC [2]. This type of carcinoma is more likely to have hematogenous metastasis, so its prognosis is better when the tumor is minimally invasive (versus extension into blood vessels, bones, lungs, etc.) [2].

Our patient was diagnosed with FV-PTC mixed with foci of cribriform morula carcinoma. Follicular-variant papillary thyroid carcinoma is a subtype of PTC that is an intermediate between FTC and classical papillary thyroid carcinoma (C-PTC). This carcinoma accounts for one-third of PTC cases and has a follicular structure lined by cells that have the nuclear features of PTC [10]. To further demonstrate its “in-between” status, one systematic study demonstrated the mean tumor size of FV-PTC was slightly larger than that of C-PTC but smaller than in FTC; in addition, fewer lymph-node metastases occurred than in C-PTC cases, but more than in FTC [10]. On the other hand, cribriform-morula has recently become its own distinct category of thyroid carcinoma [2,11]. Though rare, the cribriform-morular variant (CMV) of PTC usually occurs in young females, and it has a five-year survival rate of 90% and a 20-year survival rate of 77%, pointing to an excellent prognosis [11,12]. Though CMV-PTC is an uncommon form of thyroid carcinoma, its association with familial adenomatous polyposis (FAP) is cause for concern. Familial adenomatous polyposis is a rare genetic disorder characterized by numerous adenomatous polyps, most often lining the colon or rectum [13]. If not caught early in childhood or adolescence, it has the potential to progress into an invasive carcinoma [13]. Researchers concluded that an alteration in the adenomatous polyposis coli (APC)/β-catenin pathway, which leads to the accumulation of β-catenin, is specific to FAP-associated thyroid carcinoma [14]. Therefore, staining techniques for β-catenin in cells are used to confirm CMV-PTC. In this case, the presence of a β-catenin translocation indicated a possible association with cribriform morular carcinoma despite other features pointing to FV-PTC. This combination of tumor types led to genetic testing in our patient, which revealed DICER1-related pleuropulmonary blastoma (PPB) familial tumor predisposition syndrome. The DICER1 gene is thought to be a tumor suppressor gene, as its gene products interact to modulate posttranscriptional gene silencing [3]. Patients with DICER1 syndrome (a heterozygous germline pathogenic mutation on the DICER1 gene) have an increased risk of developing a variety of tumors, benign and malignant; examples of locations include the lungs, thyroid, kidney, head, neck, and gastrointestinal tract [3]. Because this syndrome affects several organs, the identification of tumors (such as PPB or pituitary blastoma) very early in life should initiate the evaluation for the DICER1 mutation [4].

The risk of cancer that stems from DICER1 syndrome in males and females is equal during childhood; in adulthood, females with the mutation may exhibit an increased risk due to the risk of gynecologic cancers such as ovarian Sertoli-Leydig tumors [3,4,15]. DICER1 mutations are the major cause of familial and nonfamilial PPB, which is a cancer in the lung tissue or pleura of children [16]. Individuals with DICER1 also are at risk of developing cystic nephroma and multinodular goiters [3,17]. Venger et al. describe one family’s rare case in relation to DICER1’s pathogenic variation: a pair of siblings who were both diagnosed with PTC at age 17, in addition to the brother and mother developing thyroid nodules during their childhood years. Both the sister and the mother had ovarian Sertoli-Leydig cell tumors at five years and 23 years, respectively. All carried a heterozygous pathogenic DICER1 variant, which emphasizes the importance of genetic counseling [18].

Due to the spectrum of DICER1-associated tumors, screening should be undertaken once the mutation is identified. The most common feature noted in persons harboring a germline pathogenic loss-of-function variant of DICER1 is a thyroid nodule, with more than 50% of females with this variant developing multinodular goiter in their lifetime [5]. A thyroid ultrasound is useful in looking at multinodular goiters and thyroid nodules, as one study found 32% of women and 13% of men with the pathogenic variant in the cohort were diagnosed with multinodular goiters and/or had a thyroidectomy by age 20 [5]. The literature further recommends individuals with DICER1 mutations should have a thyroid ultrasound every three years from ages eight to 40 years [19]. Imaging guidelines advise chest X-rays every four to six months from birth until age eight years (and annually from ages eight to 12 years), along with computed tomography scans at age three to six months and again at three years of age in patients with a confirmed DICER1 pathogenic variant [15]. Chest imaging is especially important, as 65% of children with PPB have a DICER1 variant [15]. In fact, PPB is the most common presentation of DICER1 syndrome. However, radiation should be used judiciously in these patients, as they are at increased risk of radiation-induced tumors. In addition, pelvic ultrasounds every six to 12 months are recommended for females aged between eight to 40 years to monitor for possible growth of gynecologic or renal tumors, such as ovarian Sertoli-Leydig tumors and cystic nephromas (both associated with the DICER1 phenotype) [15]. Abdominal ultrasounds can also screen for renal tumors. Early detection of these DICER1-associated tumors is important for treatment at the earliest possible stage. Knowledge of further risks also is gained from surveillance. For example, the discovery of PPB may initiate more vigilant and frequent screening of the thyroid; differentiated thyroid carcinomas such as PTC and FVC have been associated following the occurrence of PPB, possibly due to chemotherapy or increased radiation exposure when treating PPB [20].

There is no single treatment method for DICER1 syndrome because of its varying manifestations in different organs, so tumors must be addressed in accordance with their location. For example, treatment recommendations for PPB include surgical resection, while treating pituitary blastomas and pineoblastomas involves radiation, chemotherapy, or surgical removal [4]. Treating CMV-PTC and other PTC may involve a total thyroidectomy and radioiodine therapy [12]. Typically, radioactive iodine therapy is indicated in children with papillary thyroid carcinoma who fall under intermediate or high risk. For ATA pediatric intermediate- and high-risk patients, a TSH-stimulated thyroglobulin and diagnostic whole-body scan are generally recommended for further risk stratification and determination of treatment with 131I [7]. Our patient’s tumor was approximately a 50/50 mix of FV-PTC admixed with foci of cribriform morula carcinoma. There is some debate as to whether the cribriform morula variant is a PTC. The literature suggests the cribriform morula variant’s behavior is indolent; however, the estimated diameter of the follicular variant component was approximately 2 cm in our case. The outcome for patients with DICER1 syndrome is thus diverse and depends upon the malignancy of the tumors.

Our patient is unique because even though thyroid disease and multinodular goiters are frequent in the general population, they are uncommon in children [17]. And the presentation of DICER1 syndrome itself is rare, with most mutation carriers being unaffected [16]. The literature points to a 16-fold increased risk of PTC or FTC in individuals with DICER1 mutations but also suggests the syndrome’s contribution to familial multinodular goiters and thyroid carcinomas is small [5,17]. Therefore, a suspicion of the syndrome is not warranted unless other DICER1 phenotypes (such as PPB) appear as well, or if these thyroid phenotypes are present in childhood (as seen in the patient of this case study) [16]. There is a risk of thyroid cysts developing in childhood or young adulthood with this syndrome, as one study demonstrated that of 25 relatives with DICER1 mutations, six had non-toxic multinodular goiters [16]. Thyroid carcinomas are less likely to metastasize; hence, DICER1-related thyroid carcinomas are a low-risk subgroup [17]. Nonetheless, one study has shown thyroid nodules affect around 75% of female pathogenic mutation carriers and up to 17% of male carriers by 40 years [5]. Multinodular goiters are typically benign and can present as early as three years, while FTC and PTC presentation can begin at eight years of age and are typically malignant (but have a high cure potential) [17]. For our patient, monitoring by an oncologist and endocrinologist (with suggested surveillance imaging) is necessary to address the tumor in the thyroid, along with surveillance for other cancers that may develop with DICER1 syndrome.

## Conclusions

Our patient was found to have FV-PTC mixed with foci of cribriform morula carcinoma, a rare tumor suggesting a genetic etiology. The presence of a mix of two different tumor types, as well as immunohistochemical features, may point a pathologist towards suggesting genetic evaluation for a tumor syndrome such as DICER1. DICER1 PPB familial tumor predisposition syndrome is an autosomal dominant hereditary syndrome defined by several uncommon tumors, including PPB in the lungs, cribriform-morular thyroid carcinoma, and Sertoli-Leydig cell cancers in the genitourinary tract. This case presents an uncommon type of thyroid cancer and emphasizes the importance of a multidisciplinary team (i.e., interventional radiology, genetics, pathology, endocrinology, and oncology) when a tumor syndrome is suspected. The case also explores the diagnosis, management, and ongoing surveillance of a rare mixed-tumor type of thyroid carcinoma.
